# The current scenario of emergency care policies in Brazil

**DOI:** 10.1186/1472-6963-13-70

**Published:** 2013-02-20

**Authors:** Gisele O'Dwyer, Mariana Teixeira Konder, Cristiani Vieira Machado, Camila Paes Alves, Renan Paes Alves

**Affiliations:** 1 National School of Public Health / Oswaldo Cruz Foundation (Escola Nacional de Saúde Pública/Fundação Oswaldo Cruz), 1480, Leopoldo Bulhões Avenue, Rio de Janeiro, Postal code 21041-210, Brazil

**Keywords:** Emergencies, Pre-hospital care, Public health policies

## Abstract

**Background:**

The regulation of emergency care has featured prominently in Brazil’s federal health agenda since the 2000s. The aim of this study was to review up to the present day the implementation of the National Emergency Care Policy.

**Methods:**

The methods employed were documental review, analysis of official data and 11 interviews conducted with federal, state and local managers. The results were analyzed using Giddens’ Structuration Theory, relating the cognitive abilities of the agents to their action strategies, in view of the structural dimensions, rules and resources provided by the federal administration.

**Results:**

Federal policy for emergency care in Brazil can be divided into three stages: from 1998 to 2003, the initial regulation; from 2004 to 2008, the expansion of the Mobile Emergency Medical Services (SAMU, in Brazil); and from 2009 onwards, the implementation of stationary pre-hospital care facilities, known as Emergency Care Units (UPA). The structuration elements identified for the emergency care policy were the public health system guidelines, legislation, standards and federal financing. Significant restrictions were found such as lack of hospital beds and intensive care treatment, gaps in the information system for producing evidence for management, ineffective Management Committees, as well as a low degree of commitment among physicians to the services.

**Conclusion:**

Considering the financial constraints imposed on the SUS (Brazilian Unified Health System), emergency care was identified as a political priority with financial support. The individual actions by emergency care workers and governmental agents typified the first period of the policy, structuring the basis and producing changes in the circumstances of action. Federal strategies can be equated to the rules and resources provided to support the implementation process of the policy.

## Background

Brazil, a vast country with an estimated population of 193,946,886 in 2012 (Brazilian Institute of Geography and Statistics – IBGE data) is organized in a three-tier federative system composed of the central government, 27 states and thousands of municipalities, which reflect substantial regional and social inequalities. The main epidemiological challenge faced by the country today is the fight against chronic degenerative diseases, although infectious diseases typical of poor countries still affect a large portion of the population [1]. In addition to these two groups of illness, external causes have exerted noteworthy impact on the morbidity and mortality rates in the Brazilian epidemiological context.

In 1988, with the country in a state of transition to democracy, the *Sistema Único de Saúde* (SUS), or Unified Health System, was created and health was established as a universal right and State duty. Based on its guiding principles, the SUS is a universal, equal and comprehensive public health system. This major achievement was particularly admirable as it was driven not by governments, political parties or international organizations, but rather by the civil society and health professionals [1]. The gain from this health reform is more meaningful when considering it was developed against a neoliberal ideological background parallel to a significant expansion of private health care supported by expressive public financing.

From the 1980s until 2010, Brazil showed a significant reductions in infant mortality (from 69/1000 to 19/1000) and the fertility rate (4.35 to 1.86), as well as significantly increased life expectancy (62.6 to 72.8) [1]. Throughout this period access to health care services in Brazil has considerably improved. The SUS has surpassed the limits of urban areas and now serves the rural population, minorities and individuals with disabilities, the indigenous population and others [1].

An initial evaluation of the more than 20 years since the implementation of the SUS points to a significant increase in access to public health services over recent years [1-3]. However, the system still suffers from a highly restricted service capacity, when compared to the private health system. Out of all the main diagnostic imaging equipment available in the country, the following percentages are available for SUS use: X-Ray machines, 58.9%; CT scanners, 24.1%; MRI scanners, 13.4%; ultrasound equipment, 51%. In relation to the service type, primary care services are essentially public. Only 10.7% of the specialized clinics are public; 6.4% of diagnostic and therapeutic services are public; 77.9% of general and specialized emergency services are public; and 31.9% of all hospitals are public [1].

Since the 1990s the public system has been composed mainly of primary health care units and emergency services. The private network, on the other hand, has been comprised of specialized and hospital care. Consequently, low complexity and emergency services have been used more by lower income members of the public, who are the predominant SUS users [4]. That state of dominance has persisted with some improvements, without, however changing the situation which conditions the use and increased demand for emergency medical services.

The SUS, therefore, competes with the private sector against a backdrop of inequality and, although having the status of a universal system, it is highly dependent on private-public contracts, especially for diagnostic and therapeutic services. This arrangement of resources results in a lack of services to meet the demand, something which is unacceptable on the principle of universality. It should be highlighted that there is also a lack of health professionals, especially physicians, who split their working hours between public and private services.

A primary and emergency care-centered pattern of service use overburdens the emergency services, as revealed in a national survey of SUS user satisfaction conducted in 2002 – Brazilian Public Opinion on Health [5]. The low level of satisfaction among SUS users, particularly with the emergency services, identified in this survey, gave rise to a series of meetings between managers from all three governmental tiers, triggering hospital-focused investments. The investment strategies included QualiSUS (Investment Project in Improving SUS Quality), which entailed actions ranging from the physical restoration and renewal of health care facilities to the hospital humanization policy intended to diminish service deterioration, which results from the substantial and repressed demand for hospital care [6]. Regardless of the alleged "causes" of this excessive demand, there was consensus about the need to organize the emergency medical services in order to channel health care delivery toward a more rational and hierarchical utilization of the health resources [7-9].

Prior to investing in emergency medical services, in the early 1990s the Federal Government initiated significant investment in primary health care, which policy remains top priority to this day [10]. However, despite this significant investment there are still regional differences in terms of primary health care coverage, entangled with the difficulties in emergency medical services [11,12].

This demand for medical consultation in hospital emergency departments, despite the expansion of primary care services since 1990, reveals that hospitals remain important gateways for medical assistance, which could be related to restricted timely access to basic services, specialists and diagnostic support due to a lack of such services, as mentioned above [13].

Another important feature of the Brazilian health system is the tendency to structure health care subsystems, organized to respond to specific needs, which hinders effective coordination of the care services. However, there are specific SUS policies being set up in the form of networks, such as the emergency medical services, with the purpose of continued care [14].

## Methods

The aim of this study is to review the implementation of the emergency care policy in Brazil, considering specific aspects of the different stages of its development and the current challenges.

Analysis of documents and official data formed the methodology, as well as interviews with federal, state and municipal health care and emergency medical service managers. The interviews were conducted in the context of two research projects: the first about priority federal policies for the SUS in the 2000s [13] and the second about the implementation of the SAMU in the state of Rio de Janeiro [15]. The national-level interviewees were: 6 federal managers, selected according to the position held and period in office (2003 to 2010), including 2 Ministers of Health, 1 top-level federal secretary and 3 federal coordinators of emergency services from the General Coordination of Emergency Services (CGUE); 1 member of the National Emergency Service Steering Committee, and the representative of the Brazilian Cooperative Network for Emergencies (RBCE), given the importance of this network in representing emergency medical service workers and its participation in national policy making. In the state of Rio de Janeiro, the interviewees were the State Coordinator of Emergency Medical Services and the coordinators of the 3 SAMU units implemented by 2009. Rio de Janeiro was the state chosen for investigation due to the following characteristics: experience in mobile emergency medical services since 1986 through the Military Fire Department of Rio de Janeiro State (CBMERJ); early implementation of the SAMU with regional coverage; 2 of the 3 largest SAMU fleets in Brazil; the first Brazilian state to implant Emergency Care Units (UPA) and the highest number of UPAs in the country.

Giddens’ Structuration Theory was used as the theoretical framework for analysis of the results [16]. According to Giddens, social practices can be seen as procedures, methods or skilled techniques appropriately executed by social agents using rules and resources. Therefore, agents are largely free to take action, but are always conditioned by the structural resources available. Giddens sees social practices as structured within this duality of social object and individual action, and rejects the dominance of either extreme. Actors use their knowledgeability to create routine practices or produce changes depending on the “circumstances of the action”. These are the ways in which social and material phenomena facilitate or restrict human action. Such circumstances represent both the means and the result of the action, illustrating the duality of the structure [16].

The structure is formed by rules and resources. The normative aspect of the rules corresponds to practices from the perspective of rights and duties and the ways in which the practices can be executed. The semantic dimension of the rules corresponds to the qualitative and procedural meaning of the practices, associated to their performance. The resources are the facilities or power bases to which the agent has access and that he manipulates to influence interaction with others. These resources may be authoritative (position or office held, for example) or allocative (material) [17].

Access to the agent may come about through discursive consciousness and practical consciousness. The interviews were aimed at giving expression to the discursive consciousness of the actors and were analyzed using discourse analysis methodology [18].

## Results and discussion

The construction of federal policy for emergency medical care in Brazil can be divided into three stages: from 1998 to 2003, federal regulation; from 2004 to 2008, major expansion of the Mobile Emergency Medical Services (SAMU, in Brazil); and from 2009 onwards, the implementation of stationary pre-hospital care facilities, known as Emergency Care Units (UPA) [13].

The results of the study are presented according to these three stages, followed by a review of the current status of the policy.

### Federal regulation of emergency medical care

Work developed in the first stage produced the rules and defined the resources for the regulation of care services essential for tackling morbidity and mortality indicators. Prominent during this time were governmental agents, who prioritized that level of care, and actors of the practice, who demanded action in relation to such care.

In 2000, medical professionals belonging to the Brazilian Cooperative Network for Medical Emergencies (RBCE) reported to congress the lack of regulation for the field and thereafter a task force working in conjunction with the ministry of health established the conceptual basis for the publication of Ministerial Directive 2048 [19]. The CGUE coordinator at that time underlined the importance of this event. *“The Health Care Secretary (…) stood at the opening of congress and said that there actually was no medical emergency policy established in the Ministry and that he was open to hold discussions (…) we worked enthusiastically through congress to make a report and a national proposal to be submitted to the Ministry."* Communities of specialists (health professional networks and councils) pieced together alternatives for emergency medical service and were able to exert influence on the Ministry of Health in the early 2000s, resulting in the first regulatory standards being established for the field [13].

It was, therefore, this pressure exerted by agents over managers that instigated a cycle that produced the standards for emergency care, with the agents acting as advisors, proponents and demanders of new policy. This policy-making period featured agents manipulating authoritative resources, producing a change in the scenario, constructing rules and resources to establish the emergency care policy.

The RBCE and ministry managers maintained a tight partnership until 2005. Since then, despite not leveraging the same political influence of policy-making, the members of the network remain active and are a recognized partner group, producing information and analysis on the subject of emergency medical care.

As regards federal regulation, the publication of the Regulations for State Systems of Emergency Care in 2002 [19] served as the basis for the subsequent structuring of the policy. Upon its formulation, there was still no technical area within the Ministry of Health responsible for the emergency care policy or any financing mechanisms for its implementation [13]. The National Emergency Care Policy (PNAU) [20] was established in 2003 with the creation of a new management organization within the Ministry of Health, the General Coordination of Emergency Services (CGUE). The creation of the CGUE within the Ministry of Health in 2003 gave fundamental support for the effective implementation of the emergency care management policy [21]. The milestone targets of the PNAU were federal financing, regionalization, staff training, management through an emergency care committee and the expansion of the network [21].

This period can be identified as triggering a new system of emergency medical services, which has since given priority to pre-hospital care.

Table 1 lists the most important rules that regulate emergency care policy.

**Table 1:** Major federal ordinances regulating emergency care policy

**Federal ordinances/Year**	**Content**
Ordinance 2048/2002	Regulates Emergency State Systems; establishes its principles and guidelines, sets standards, operating criteria, classification and registration of emergency hospitals.
Ordinance 1863/2003	Enacts Federal Policy for Emergency Care (PNAU), to be implemented in all federal units, according to federative levels autonomy.
Ordinance 2072/2003	Creates Emergency Care National Management Committee and defines its powers and responsibilities.
Ordinance 1864/2003	Implements Pre-hospital Care Mobile Service created by Federal Policy for Emergency Care, in all municipalities and regions of Brazil: SAMU - 192.
Ordinance 3125/2006	Enacts QualiSUS program and establishes its jurisdiction. Sets guidelines to structure and organize emergency care actions focusing on pre-hospital and hospital care.
Ordinance 2922/2008	Establishes guidelines for Emergency Care Units implementation; Defines conditions, responsibilities and prerequisites for setting up these facilities within emergency care networks.
Ordinance 1600/2011	Reformulates Federal Policy for Emergency Care and creates Emergency Care Network.
Ordinance 2648/2011	Reviwes guidelines for Emergency Care Units implementation.

### Implementation of the SAMU

The Mobile Emergency Medical Service was defined as the first component of the PNAU to be implemented and its expansion consolidated the second period of evolution of the emergency care policy in Brazil. The French SAMU model was used as the template for its formulation.

The historical origin of ambulance emergency services actually dates back to Napoleonic France. In 1960s France the *Service d’Aide Médicale d’Urgence* or SAMU was created; an emergency medical service provided through ambulances connected to Emergency Call Centers, reached through a unified telephone number available to the whole population. These Centers coordinated the first response vehicles and maintained a shared communication system with the Fire Department operation centers [22].

Using an alternative model, the United States also heavily expanded emergency pre-hospital care from the 1960s onwards, in order to ensure safe and quick transportation of patients to hospitals. In this model, patient transport is executed by professionals who are trained according to federal regulations [23].

In Brazil, this US model of ambulance medical care began to be used by the Military Fire Department (CBM) in the late 1980s, with such professionals working as emergency medical technicians and providing care for traumas. The state of Rio de Janeiro was pioneer in this mode of care and has remained a benchmark by virtue of other emergency care initiatives it has developed, as shall be seen throughout this article.

In the years that followed some states and municipalities took the initiative to implement their own emergency medical services, giving rise to the first Emergency Medical Services. However, up to that point, Brazil had no nationally-regulated pre-hospital care system.

Implementation of the Mobile Emergency Medical Service – SAMU 192 – [24] began in 2003, however it was already foreseen in GM/MS Ministerial Directive 2048, of November 5, 2002. This was defined as the first step of implementation of the National Emergency Care Policy (PNAU) in virtue of its strategic potential for organizing the local and regional flows of comprehensive emergency care. At the time there was strong agreement for its implementation within the ministry and the federal government, as well as support from organized groups such as the RBCE, as one SAMU manager suggests: *“The president displayed idealism when he said: I want this for the country”*[13].

SAMU-192 represents a nationally standardized medical service model, which provides 24/7 emergency care at people’s homes, work places and on the roads. It has the goal of ensuring care, adequate transportation and routing of patients to an SUS-integrated service. Emergency response is engaged through a toll-free phone number (192) from anywhere in Brazil. Calls are answered by a Medical Regulation Call Center, organized in a regionalized and hierarchical manner, which defines the most suitable response, whether that be specific instructions or the dispatch of a team to the emergency site.

The implementation of the SAMU as the first stage of the policy was advocated based on the argument that the Medical Regulation Call Centers are key elements to the organization of comprehensive emergency medical care, as they can provide the function of observation posts of the health care system networks. The medical regulators are essential players in the SAMU as they have the prerogative to regulate access to the care service, thus making them public health authorities.

The study conducted in the state of Rio de Janeiro revealed the fragile position of these professionals in light of their duty as public health authorities, as explained by the state coordinator of emergency care: *“We are trying to make the medical regulator into a manager. If the doctor makes little fuss of his managerial role, then the system suffers”*[15].

It may be asserted that unstable employment relations during SAMU regulation in Rio de Janeiro did little to facilitate the professionals’ actions, hindering these regulators in their deployment of service resources against a backdrop of limited state cooperation in the hospitals.

The regulator’s function is further destabilized by two structural constraints in the system: a lack of hospital beds for emergencies and a lack of ambulances and ambulance crews.

The ambulances and ambulance crews constitute a structuring resource of the SAMU. The main types of ambulance available in the SAMU are: Basic Support Units, which have few technical resources and a crew limited to emergency medical technicians; and Advanced Support Units, with intensive care resources and a crew composed of a physician and nurse. The federal standard recommends the allocation of one Basic Support Unit for every 150,000 inhabitants and one Advanced Support Unit for every 400,000 inhabitants. These proportional parameters have been questioned and the SAMU coordinators negotiate their needs accordingly, as the following account reveals:

*“I estimated the need for Advanced Support Units, but I know that it’s no use getting them all because I won’t be able to assign all the medics. Physicians don't want to work in ambulances for long due to the violence, the low salary (…)"*[15]. Data relative to the SAMU services – like response time for ambulance dispatch, conformity of the ambulances, access to hospitals – would be useful for action planning toward expanded and qualified access to emergency medical services in Brazil [13]. Currently such information is not regularly available to all levels of management.

Another feature that has affected emergency care in the state of Rio de Janeiro and, especially, in the state capital, is the disproportionate number of hospital units in relation to primary care. The public hospital network of Rio de Janeiro state is split into municipal, state and federal government-managed units, as well as those run by philanthropical organizations and universities, which fact has hindered the integration of the emergency medical services. The study in Rio de Janeiro state indicated the restricted access to hospital beds, which are not systematically available to the network manager: *“The hospitals are not cooperative with their beds because in some services the bosses think they are the perpetual owners, and for years have selected the patients who occupy those beds"*[15]. This power over hospital beds that should be available for the medical regulators is a direct result of the distinct hospital managements found in Rio de Janeiro.

Another planning instrument established in the National Emergency Care Policy is the formation of steering committees at municipal, regional, state and national levels, responsible for coordinating between managers, formalizing agreements on priority actions and analyzing the performance indicators proposed by the policy [25]. A member of the National Emergency Care Steering Committee described the scope of power of the committees: *“The steering committee should have an active life, it has to be dynamic because it has to have penetration; it’s one of the medical regulator’s work tools. If the committee works with the services, he, the medical regulator, will no longer need to negotiate spaces on demand".*

This piece of legislation [25] also recognizes the need to train SAMU professionals in providing telemedicine care in the role of system regulators. This training also aims to overcome the absence of emergency medicine as a medical specialty in Brazil.

Implementation of this policy, however, is developed quite differently throughout the country, depending on the specific context, the degree of state and municipal adherence to the policy, the local health system structure and the organization in place prior to the service network.

There was also a high degree of variation in the transition process from emergency medical services traditionally performed by the Military Fire Department to the SAMU-192 strategy. There were several forms of coexistence between these two models, according to specific arrangements at each location [26].

The number of SAMU units in Brazil increased progressively between January 2004 and July 2009, slowing down since. As regards the type of coverage, the big cities displayed early adherence to the strategy, but by 2008, the number of regional SAMU units had exceeded the municipal ones. This seems to reflect the Ministry of Health’s concern to subsequently encourage the participation of the States and regional organization of SAMU units in order to include smaller municipalities in the emergency medical service networks. This phenomenon is illustrated by the statement of the CGUE coordinator, also in 2008: *“No SAMU projects will be initiated from now on apart from regional ones"*[13]*.*

However, as is to be expected in a federative set-up, there are marked differences between the states in terms of SAMU coverage and implementation characteristics [13].

As a recent initiative on the national scenario, there is still very little in the way of literature about the SAMU. Nevertheless, the low quality information system used by SAMU units, with software provided by the Ministry of Health, would seem to represent a significant obstacle for the development of indicator-based actions. This handicap was pointed out in a previous study [15] and confirmed by a SAMU coordinator: *“The indicators we use are awful because of the system. (…) the reports it produces are really bad (…) the fact that everyone wants to have their own system, it looks like it might work, but it is a huge step back. How am I supposed to formulate national investment policies?"*

Local experiences have generated studies that contextualize this service, revealing significant diversity in the models adopted in Brazil and indicating major challenges to be tackled.

In Rio de Janeiro, pioneer state in pre-hospital care provided by the Fire Department, the first regionalized SAMU of Brazil, named Metropolitan II, was installed in 2004 to service 7 municipalities and almost 2 million people. In this case, the Regional Emergency Care Management Committee was fundamental in guaranteeing equal care distributed in a region where the poorest municipality was the one that consumed the most SAMU resources [27]. After three years of operation, the committee’s role became undermined, with restricted funding identified as the main coercive factor for this SAMU [15].

A recent study describes the SAMU as a complex system due to the unstable nature of the work, the variety of demands, the uncertainty involved in the required interactions and limited understanding among professionals of the variables involved in their work and other motives [28].

Another study, conducted in five large cities with high rates of violence, indicated SAMU implementation as a positive action in tackling urban violence [26].

Several studies have identified the lack of ambulances and professionals, as well as restricted integration with the network for access to hospital beds, as limiting factors [15,27,29,30].

In relation to the types of demand presented to the SAMU, clinical demands are predominant, including demands not regarded as emergencies that could be resolved through primary care [15,31,32].

SAMU staff training was reviewed in a study that indicated a low degree of adhesion to continuing education programs among its medical professionals. The authors suggest that staff education and training should be provided during their shifts and be counted as hours worked in order to ensure adhesion to such programs [29].

These studies portray a situation in which complex demands, an insufficient primary and hospital care network, weak agreements, low-quality staff training and unequal access may not be restricted to the experiences studied, suggesting the need for new investigation issues.

A proposal currently exists to expand the SAMU to cover 100% of the population by 2018. It should be highlighted that the population coverage of the emergency medical service (65%) already exceeds that of primary health care in Brazil (53%) (data from the Ministry of Health website http://www.saude.gov.br in October 2012). Nonetheless, a strategy has yet to be defined to ensure the efficacy of the SAMU information system and to tackle the weakness of the Steering Committees at the various management levels.

### Stationary pre-hospital care, a new strategy for emergency care

After the first two stages of the National Emergency Care Policy implementation - initial regulation and the expansion of the SAMU - the process to install and prepare to standard the Emergency Care Units (UPA) began to unfold in 2008 [33]. Raising this instance to priority level, the federal government established standards and provided funds for its development. The basic requirement was that these units were to be installed strategically in line with the organization of the comprehensive emergency care networks and must be linked to the SAMU.

Defined as facilities of intermediate complexity, between primary care and the hospital system, the UPAs are meant to establish an organized network of emergency care, with mutual agreements and flows to ensure patient reception, allowing for counter-referrals to the service network, when applicable. The UPAs are open to the public 24/7 and serve as a qualified rearguard for the stabilization of SAMU patients. When a patient’s complaint is not resolved within 24 hours' observation, he/she should be referred for admission to one of the network hospitals. [33,34]. Currently 90% of the UPAs installed in the country are concentrated in just seven states, with the highest number of UPAs in the state of Rio de Janeiro.

Rio was also the first state to implement 24-hour UPAs, the stationary pre-hospital emergency unit foreseen in the PNAU. The fast expansion of these facilities in the municipality of Rio de Janeiro was justified by the substantial deficit in primary care, with the aim of reducing the demand on hospital emergency departments. The most suitable gateway to the SUS is through structured primary care, where multidisciplinary teams can follow patients’ care and development. However, in the absence of regulation and integration between the most effective services, all levels of emergency care are left with the task of absorbing the demands [21].

### The challenges for emergency care

As regards the future development of the emergency care policy, the Ministry of Health and the General Coordination of Emergency Services have proposed in 2012 the creation of an Emergency Care Network (RUE) [35], with the purpose of coordinating and integrating all health care equipment in order to expand and enhance humanized and comprehensive user access to prompt emergency care. The following actions are proposed to achieve this: preventive care, health promotion and primary care provided through accident and violence prevention centers, in addition to the installation of clinical observation and stabilization rooms where there are care gaps; the creation of a national health taskforce to provide support in risky and vulnerable situations and disasters involving multiple victims; regionalized expansion of the SAMU up to 100% coverage; UPA expansion; investment and improvement of hospital emergency departments, covering hospitals with over 100 beds which are regional references and offer priority services (cardiovascular, neurosurgery and trauma/orthopedics); incentives for rearguard wards and intensive care units, and 100% coverage for home care. The proposal will follow a staggered implementation schedule from 2012 to 2018.

**Figure 1:**
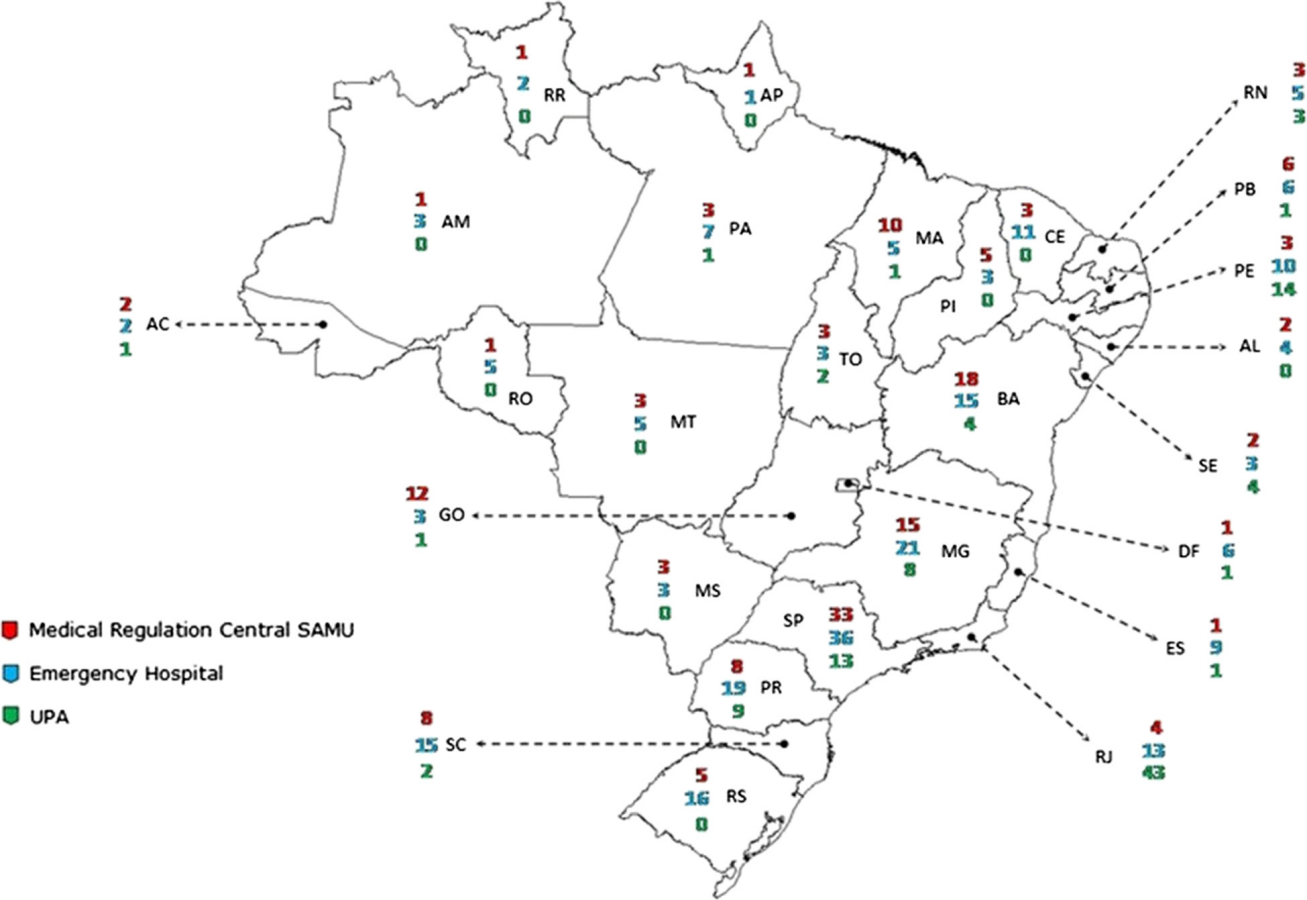
**Emergency care units per type in each state in 2011.** Source: Ministry of Health – Emergency Care General Coordination. States in alphabetical order: AC: Acre; AL: Alagoas; AM: Amazonas; AP: Amapá; BA: Bahia; CE: Ceará; DR: Distrito Federal; ES: Espírito Santo; GO: Goiás; MA: Maranhão; MG: Minas Gerais; MS: Mato Grosso do Sul; MT: Mato Grosso; PA: Pará; PB: Paraíba; PE: Pernambuco; PI: Piauí; PR: Paraná; RJ: Rio de Janeiro; RN: Rio Grande do Norte; RO: Rondônia; RR: Rorâima; RS: Rio Grande do Sul; SC: Santa Catarina; SE: Sergipe; SP: São Paulo; TO: Tocantins.

These actions have the value of addressing emergency care in a comprehensive manner, but fail to overcome the lack of beds for emergency care in Brazil and the management issues, another weakness of the SUS, as illustrated by one State Coordinator: *“In relation to doctors, we have two problems, quantitative and qualitative deficiencies"*[15] (Figure 1).

## Conclusions

The National Emergency Care Policy has been another step on the way toward improving the quality of hospital care, as hospital-based emergency care is a major source of problems.

The federal strategies for implementation of the policy can be equated to the rules and resources provided to support this implementation process.

The first stage of the policy implementation was heavily influenced by emergency health care workers who, together with the government, produced a foundation standard for the policy and achieved a managerial mechanism within the Ministry.

The second stage of implementation prioritized the SAMU, due to the lack of regulation of such care and its inexistence throughout much of the country. Although the idea of prioritizing just one part of the emergency care policy was a fragmented investment proposal, the outcome has been very positive.

The implementation of the third stage of the policy, focusing on the UPA still requires more extensive analysis.

The study aimed to describe the three stages of the National Emergency Care Policy by means of documental review and discourse analysis of the accounts given by stakeholders. A case study was also conducted in one Brazilian state – Rio de Janeiro – which has played an important role in implementing the emergency care policy.

Limitations of the study include the fact that it was based predominantly on the review of official documents and the discursive consciousness of the agents and it was not possible to gain access to the performance indicators for the emergency care services. Furthermore, the state of Rio de Janeiro does not necessarily provide a fair representation of the experiences throughout Brazil, given the heterogeneous nature of the country and of the implementation of the policy in local contexts. This particular state is renowned for organizational difficulties in primary care and the existence of a large number of public hospitals linked to the three governmental spheres, yet suffers from significantly limited relations within the service network.

Nevertheless, the study allowed for the identification of constraints in the public health system of a more general nature, which have an effect on the development of the policy and the organization of emergency care. Underfinancing, a serious restriction that affects the SUS is partly a result of the competition with the private sector; this dualism has structured the health system in Brazil. There has been a tendency to overcome underfinancing for emergency care, with the federal government using financial induction to implement the policy. For instance, in the RUE proposal [35] for UPA expansion, a target is set of 1,096 UPAs by 2018, without specifying the criteria for this expansion. If this fast-track expansion process, with federal financing, is not guided by technical criteria then this could compromise the conditions and efficacy of the new unit implementations.

The structural development of the SUS in a context of dependency on, and competition with the private system has led to weakened attachment and commitment among medical professionals to the public service. This entails huge difficulties in health care facilities, but has been particularly restrictive for the job of the medical regulator. The expressive expansion of the SAMU, the UPA and primary care has led to competition for SUS doctors.

A diverse group of actors has influenced this policy and enabled its construction: federal government officials (including the President of the Republic from 2003 to 2010), state and municipal government officials, professional corporations (firefighters) and professional groups (RBCE).

Analysis of the results, according to Giddens’ Theory of Structuration, showed that the financing and regulation by federal standards have been structuring elements for the emergency care system in Brazil. The doctors from the Brazilian Cooperative Network for Emergency Care, followed by federal managers, were the key drivers of this process and ensuring change in the national emergency care policy.

The majority of the international literature published on emergency care addresses care in the hospital setting. The analysis performed in Brazil differs little from that produced in other countries. There is a huge issue with overcrowding, with its respective control strategies. The authors focus mainly on the management strategies to improve hospital care [7]. Despite the broadened scope of the Brazilian policy to encompass various components of the care service, hospital care remains very fragile. A lack of beds for patients suffering from acute disease and beds for Intensive Care Units (ICU) constitute a structural characteristic of the SUS that must be tackled.

The aim of the emergency care network is to drive changes in national policy. Further studies are required to address the integration of the system in a more systemic manner and to investigate and describe the distinct experiences throughout the various Brazilian states and municipalities.

## Abbreviations

CBM: Military Fire Department (Corpo de Bombeiros Militar); CBMERJ: Rio de Janeiro Military Fire Department (Corpo de Bombeiros Militares do Estado do Rio de Janeiro); CGUE: General Coordination of Emergency Services (Coordenação Geral de Urgência e Emergência); PNAU: National Emergency Care Policy (Política Nacional de Atenção às Urgências); RBCE: Brazilian Cooperative Network for Emergencies (Rede Brasileira de Cooperação em Emergências); RUE: Emergency Care Network (Rede de Atenção em Urgência e Emergência); SAMU: Mobile Emergency Medical Service (Serviço de Atendimento Móvel às Urgências); SUS: Unified Health System (Sistema Único de Saúde); UPA: Emergency Care Units (Unidades de Pronto Atendimento); UTI: Intensive Care Unit (Unidade de Tratamento Intensivo).

## Competing interests

The authors declare to have no conflict of interests.

## Authors’ contributions

GO and MTK made substantial contributions to the conception and design of the study, data collection, analysis and interpretation and wrote the manuscript. CVM made substantial contributions to the conception and design of the study, data collection, analysis and interpretation. RPA and CPA made substantial contributions to the data collection, analysis and interpretation. All the authors have read and approved the final manuscript.

## Authors’ information

GO e CVM are researchers and MTK, CPA e RPA are students belonging to the research group that investigates the “Formulation and implementation of public policies and management of health systems theoretical and methodological approaches in public policy analysis”.

## Pre-publication history

The pre-publication history for this paper can be accessed here:

http://www.biomedcentral.com/1472-6963/13/70/prepub
